# Ranking Landscape Development Scenarios Affecting Natterjack Toad (*Bufo calamita*) Population Dynamics in Central Poland

**DOI:** 10.1371/journal.pone.0064852

**Published:** 2013-05-29

**Authors:** Kamila W. Franz, Jerzy Romanowski, Karin Johst, Volker Grimm

**Affiliations:** 1 Centre for Ecological Research Polish Academy of Sciences, Dziekanów Leśny, Poland; 2 UFZ, Department of Ecological Modelling, Helmholtz Centre for Environmental Research – UFZ, Leipzig, Germany; 3 Museum and Institute of Zoology, Polish Academy of Sciences, Warsaw, Poland; 4 Institute for Biochemistry and Biology, University of Potsdam, Potsdam, Germany; Texas Tech University, United States of America

## Abstract

When data are limited it is difficult for conservation managers to assess alternative management scenarios and make decisions. The natterjack toad (*Bufo calamita)* is declining at the edges of its distribution range in Europe and little is known about its current distribution and abundance in Poland. Although different landscape management plans for central Poland exist, it is unclear to what extent they impact this species. Based on these plans, we investigated how four alternative landscape development scenarios would affect the total carrying capacity and population dynamics of the natterjack toad. To facilitate decision-making, we first ranked the scenarios according to their total carrying capacity. We used the software RAMAS GIS to determine the size and location of habitat patches in the landscape. The estimated carrying capacities were very similar for each scenario, and clear ranking was not possible. Only the reforestation scenario showed a marked loss in carrying capacity. We therefore simulated metapopulation dynamics with RAMAS taking into account dynamical processes such as reproduction and dispersal and ranked the scenarios according to the resulting species abundance. In this case, we could clearly rank the development scenarios. We identified road mortality of adults as a key process governing the dynamics and separating the different scenarios. The renaturalisation scenario clearly ranked highest due to its decreased road mortality. Taken together our results suggest that road infrastructure development might be much more important for natterjack toad conservation than changes in the amount of habitat in the semi-natural river valley. We gained these insights by considering both the resulting metapopulation structure and dynamics in the form of a PVA. We conclude that the consideration of dynamic processes in amphibian conservation management may be indispensable for ranking management scenarios.

## Introduction

Due to human impact many species are threatened with extinction. The main goals of conservation biology are to identify which species are endangered, which factors drive species to extinction and to develop strategies to prevent extinctions. Because of the scale and speed of species extinctions conservationists require methods that facilitate decision making in the light of scarce data. Important tools often used by policy makers include habitat models [1] and population viability analysis (PVA) which are used to assess threats and to identify the most suitable management scenarios for a given species [2]. However, it is often challenging to decide which tool is most appropriate for a particular problem and to what extent the ranking of management scenarios depends on the choice of the tool. In this study, we therefore contrast scenario ranking resulting from habitat modelling with that from population dynamic modelling.

We addressed this issue by focusing on the impact of real landscape management scenarios on the Natterjack toad (*Bufo calamita Laurenti*, 1768) in Poland. This more applied approach allowed us to examine possible conservation implications for amphibian species in general and for natterjack toads in particular. Amphibians belong to the most vulnerable class of vertebrates, with 32% of all known species under threat [3], [4]. Multiple causes of the decline have been identified, including habitat loss and fragmentation [5]–[7], climate change [8] as well as *chytrid fungus (Batrachochytrium dendrobatidis)* [9]. Natterjack toads have been shown to suffer from all the above mentioned factors (e.g. [5], [10]). However, information on the distribution and demography of the natterjack toad in Poland is extremely sparse. Collecting such data, especially for species with cryptic life stages and wide population fluctuations can be very costly and time consuming [11].

This lack of systematic field studies makes it difficult to assess the population status and prospects of the Natterjack toad. In particular, it is challenging to identify specific threats to population viability and to assess alternative management scenarios for species protection. There are very few conclusive studies on the impact of road-crossing mortality on dynamics of amphibian populations [12] so we gave special attention to the impact of road mortality on natterjack toad populations. Therefore, we additionally investigated if this problem could be addressed by a modeling approach. Based on the case study of natterjack toad, we show that despite sparse local data, a meaningful ranking of management scenarios is possible by combining landscape information and a population viability analysis, which can be based on demographic parameters extracted from the literature.

Due to increased human pressure, our study region is rapidly undergoing changes in land use and spatial development. The impact of possible landscape development scenarios on biodiversity was addressed by the project VEDI [13], [14] and a multi-stakeholder planning approach was followed. Multiple species were considered using the generic habitat modelling software tool LARCH, which allows the identification of “key patches” (large patches with a stabilising role in habitat networks, [15]). However, in the multiple species approach single species were represented rather coarsely based on the local experts’ knowledge, and population dynamics were ignored altogether. Therefore, here we augment existing assessments of management scenarios by extending the original expert model approach (LARCH) by a population viability analysis (PVA) of a single species – the natterjack toad, using the software tool RAMAS GIS [16].

We did not aim to precisely predict current or future population sizes and patch occupancy. Instead our aim was to assess and compare four alternative management scenarios concerning their suitability for natterjack toad protection. We tried to find a solution to the common problem that in the face of rapid landscape changes a decision about landscape development has to be made within limited time and resources. Such a situation might favor the use of simple habitat modelling over the more data demanding PVA. However, we will show that reliable ranking of management scenarios may require consideration of dynamics processes by a PVA instead of focusing on the habitat amount alone. We will show that this is possible even if data on these processes are scarce.

## Materials and Methods

### The Study Area

**Figure 1:**
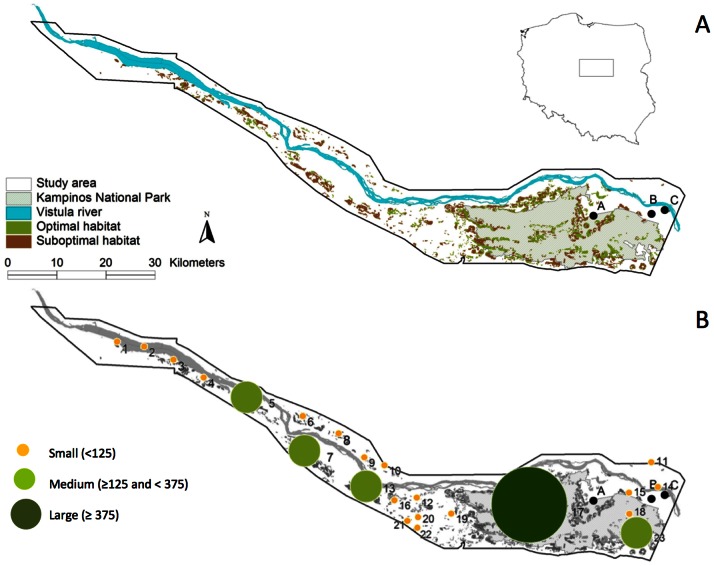
Study area and metapopulation structure. (A) Study area of the Vistula river valley in central Poland. Optimal habitat for the natterjack toad is marked green and suboptimal habitat is marked brown. Location of the study plots is marked: A – “Brzozówka”, B – “Sadowa”, C – Kępa Kiełpińska”. (B) Metapopulation structure for no change scenario. Sizes and colours of circles indicate sizes of populations (numbers of individuals).

The study area is characteristic of the Vistula River and many other central European lowland rivers with the valleys formed during the Pleistocene glacial periods. It covers a 135 km long section of the valley from Warsaw to Włocławek in central Poland with an area of about 1 545 km^2^ (Figure 1a). The river is only partially transformed by the Włocławek Dam Reservoir located in the lower course of the study area. Segments of the river show a braided pattern with many islands and sandy bars. Flood-control embankments (protective dikes) constructed at distances up to several hundred metres from the main channel limit the inundated area. The area between the dikes is covered by diverse, semi-natural vegetation: permanent meadows, shrubs and riparian forests. Floodplains contain a network of diverse aquatic sites with numerous meanders, former channels and oxbows of various sizes. The study area represents a landscape of low intensity use, with a high nature value and a high level of biodiversity [17]. Management in the valley predominantly involves agriculture: arable lands, gardens and orchards cover 33% of the area, grasslands (mostly meadows of low intensity use) cover 17%, and forests cover 34% [13]. Approximately one third of the study area is protected by 20 small nature reserves, Gostynin-Włocławek landscape park (390 km^2^), and Kampinos National Park (385 km^2^). Two areas were included in the NATURA 2000 Network: the Middle Vistula Valley (SPA Pl083) and Kampinos Forest (SPA Pl084 and SAC 158) (Figure 1a).

### Management Scenarios

Potential threats and expected or possible management strategies identified for the study area were represented in several scenarios that were developed in consultation with various stakeholders [13]. Detailed maps were prepared for these scenarios, delineating potential changes in vegetation, hydro-technical regulation of the Vistula River, and the network of roads in the study area. For our analysis we considered the following four management scenarios as opposed to the “no change” scenario illustrating present conditions in the study area [13], [14]. In addition to the general characteristics described below, these scenarios differ regarding the amount of specific habitat and landscape elements relevant for *B. calamita* as well as several demographic rates used in the model, which are summarised in Table 1 and Tables S1a and S1b.

**Table 1:** Habitat amount and landscape elements relevant for Natterjack toad characterising the four management scenarios, shown as per cent change of “no change” scenario.

	No change	Infrastructure development	Reforestation	Grassland restoration	Renaturalisation
**Wintering habitat**	[km^2^]	% change			
juvenile pine-birch forest	1.54	0	38	0	0
pine thicket	57.25	77	−22	2	78
juvenile pine forest	44.36	−95	82	3	−96
**Optimal breeding habitat**					
Wet marsh marigold meadows	71.34	0	0	0	−8
Rich pastures with *Cynosurus*	17.17	−9	−1	0	14
**Suboptimal breeding habitat**					
Complexes of segetal communities	75.13	0	−70	0	0
Oat-grass meadows	116.63	−1	−4	0	5
**Landscape elements**	[km]	% change			
Major road length	186.922	21	0	0	−100
Minor road length	1 108.106	100	0	0	−100

### Infrastructure Development

The objective of this scenario is to increase economic efficiency in transport and energy production. This scenario is characterised by maximal river regulation and the development of infrastructure. Main elements are the construction of two new dams, removal of all trees in the area constrained by the dikes, and development of other infrastructures such as roads, dikes, motorways etc. Construction of new dams and reservoirs in Wyszogród and Płock will cause disturbance of wildlife movement. Flooding the area between the dikes and in the lower and upper course of the river will result in disappearance of several nature reserves in the area and changes in the plant communities (e.g. reduction of willow-poplar alluvial forests). Construction of an intensive road and railway network will form often impassable barriers for dispersal of many animal species.

### Reforestation

This scenario is characterised by conversion of low-productivity agricultural fields into forest plantations and natural pine forest succession. The Programme of Rural Areas Development fund is designed for afforestation of areas which are not state property including agricultural areas in Kampinos National Park. Afforestation is planned in areas which are least suited to intensive agriculture (arable lands, meadows, and pastures), while reforestation is planned for wastelands in pine forests, mixed oak-pine forests and willow-poplar alluvial forests. Planned changes are also connected to transformations of forest age classes, e.g., a pine thicket evolves into a stick stand and then into an undefined pine forest. Willow plantations will be limited to the floodplain, which is the natural habitat of willow shrubs.

### Grassland Restoration

This scenario presents only moderate changes to present conditions. The main issue addressed here is the abandonment of mowing and grazing practices due to habitat fragmentation and sale of the land to non-farmers. Therefore the main objective is to restore and protect the biodiversity of grasslands by supporting the small-scale farming and traditional grazing through the implementation of agro-environmental programmes. Such programmes will focus on areas of low intensity use of meadows (mown once or twice) and of lowland pastures spread over most of the study area except for the Kampinos National Park. The aim is the preservation of existing grasslands and maintainance of the existing mosaic of grasslands, arable lands, gardens and orchards as a profitable form of land-use for farmers. It is expected to convert ruderal vegetation (by mowing) to more valuable grassland.

### Renaturalisation

This scenario is, from the conservation biologists’ point of view, a “brave vision” for Vistula valley renaturalisation aiming to restore the natural river with minimal anthropogenic impact. Its main elements include the elimination of road impacts by constructing fauna passages on existing roads, removing some of the dikes (where possible), dismantling the present dam at Włocławek, removal of some of the settlements in the river valley, and enhancement of natural succession within the floodplain. Removal of dikes along the river will result in a higher probability of flooding and in a higher risk of grain production leading to a reduction of arable fields. Decreasing the height of the Włocławek dam and protecting the banks against surface erosion will result in changes to the water flow regime in the region, requiring technical modifications of river banks. As a result new islets and sandy areas within the riverbed will be formed; also previously flooded areas will be uncovered. Promotion of meadows, pastures, and natural succession processes in the Vistula valley will result in a reduction of arable lands.

### Metapopulation Structure

Field observations of breeding amphibians were conducted in three plots (“Brzozówka” −10.5 km^2^, “Sadowa” −6 km^2^, “Kępa Kiełpińska” −18 km^2^) in the eastern part of the study area from April to June in 2005, 2007 and 2008 (Figure 1a). Natterjack toads were present at two study plots: “Kępa Kiełpińska” (one observation in 2005) and “Brzozówka” (22 observations in 2005, 25 in 2007, 21 in 2008). All breeding habitats of natterjack toads were recorded in agricultural areas, mostly low intensity use meadows. The parameterisation of the metapopulation model was based on landscape and vegetation characteristics of these sites and additional data on natterjack toad occurrence in the study area [18] as well as unpublished data from several zoologists (see Acknowledgments and Tables S1a and S1b).

We used digital maps presenting detailed actual vegetation at a scale of 1∶25000 [19]. At the landscape scale, pond presence is associated with specific meadow and pasture communities. Within these communities, four complexes were identified: (1) wet marsh marigold (*Caltha palustris*) meadows and (2) rich pastures with *Cynosurus* as optimal breeding sites, (3) oat-grass (*Arrhenatherum*) meadows, and (4) complexes of segetal communities on poor habitats as suboptimal breeding sites [20]. However, estimating the potential presence of the species should include not only the breeding habitat but also the availability of wintering habitat in the vicinity [21]–[24]. Juvenile indefinite pine forest, pine thicket and juvenile pine-birch forest (commonly associated with sandy hills in the study area) were defined as natterjack wintering habitats [25], [26] (Table 1).

Only suitable breeding habitats that were located no further than 500 m away from a wintering habitat [27], [28] were selected and defined as separate layers for each scenario in ArcView3.3 and Spatial Analyst 2.0 [29], [30]. For each layer a single ASCII grid map was created (250 m cell size grids). To obtain the patch structure, maps were analysed in the generic PVA software RAMAS GIS 5.0 [16], where values were assigned for optimal and suboptimal habitat (1 and 0.5 respectively; [13]). All cells in the distance of 6.5 cells (cell size 250×250 m) belonged to one patch (within migratory range reported by [31]). Multiplying habitat suitability value by maximum density of 50 individuals per km^2^ [32] defined the carrying capacity of each patch which corresponds to the initial conditions of the metapopulation model (Table S1a).

### Metapopulation Dynamics

To obtain detailed demographic parameters we conducted a broad review of the existing literature on natterjack toad. To assess the different development scenarios, we determined mean metapopulation abundances and extinction probabilities after 100 years from 1 000 simulation runs.

Within RAMAS GIS, we created an age- and sex-structured stochastic model with seven age classes for both males and females, corresponding to the maximum reproduction age assumed in the model [24], [33], [34]. In our model, individuals of both sexes are able to reproduce throughout their adult life starting in the 3rd year of life [20], [24], [27], [34]–[36]. The mating system was set to polygynous with a maximum number of three males per breeding female [37]. The sex ratio (F:M) was set to 1∶1.3; females produce 2.3 new females and three males [24] with a breeding success of 90% (number of females breeding each year; [36]. It is known that juveniles do not come back to the breeding sites until they reach maturity [27]. Therefore we included density dependence in the form of ceiling carrying capacity only for adult individuals [32] (Table S1a).

To estimate adult survival rates we used the annual mortality values reported in Germany by Stephan et al. [24] for medium weather conditions, assuming that breeding males’ survival rates are reduced by 5% to account for the higher vulnerability to predation during mating season [38]. Road infrastructure has been increasingly identified as a direct cause of mortality [12], [39], therefore to reflect the differences between scenarios (Table 1), in the infrastructure development scenario we assumed an additional road mortality of 6% affecting all adult individuals during their movement to and from breeding ponds [10], [40], [41]. The changes assumed in the renaturalisation scenario (complete elimination of road impact) have the opposite effect on natterjack mortality rates (6% decrease) (Table S1b).

A mean dispersal distance of 2 km was used for the dispersal function [22], [36], [42]. A maximum dispersal distance set to 10 km [42], [43] combined with a dispersal proportion of 0.2 of all individuals [35], [44] (Table S1a) allowed us to obtain dispersal rate between patches that were similar to those reported by Marsh and Trenham [42].

### Sensitivity Analysis

We changed, one after the other, each parameter value by ±20% compared to the standard values and recorded the resulting changes in carrying capacity and metapopulation structure. We choose these large parameter changes to make sure to capture the sensitivity of model predictions to the rather high uncertainty in model parameters. The price for this is that with such large changes, nonlinearities and interactions between parameters can mask differences in sensitivities of individual parameters. In our analyses, though, we saw no indication that this was the case for our model. To test the sensitivity of the scenario ranking, we used the simple outranking method (based on PROMETHEE [45]; described in detail by Drechsler et al., [46]). With this method, it is possible to determine how well the different rank orders produced by sensitivity analysis correspond with the single rank order from the original dataset. In this method ranking is done by comparing pairs of values to decide which of the two is preferred. All parameter combinations received equal weights and the preferred action receives one point. Next, the points are summed to obtain a total preference matrix containing sums of preference values for each pair of scenarios. These preference values equal the number of times one scenario ranked higher than the other scenario. Row sums measure how many times a target scenario was preferred compared to other scenarios and column sums indicate how often another scenario was preferred compared to the target scenario. Additionally to the general sensitivity analysis we investigated the impact of road mortality on the ranking of scenarios considering that there is particular uncertainty and potential sensitivity in this parameter.

## Results

### Metapopulation Structure and Carrying Capacity

**Figure 2:**
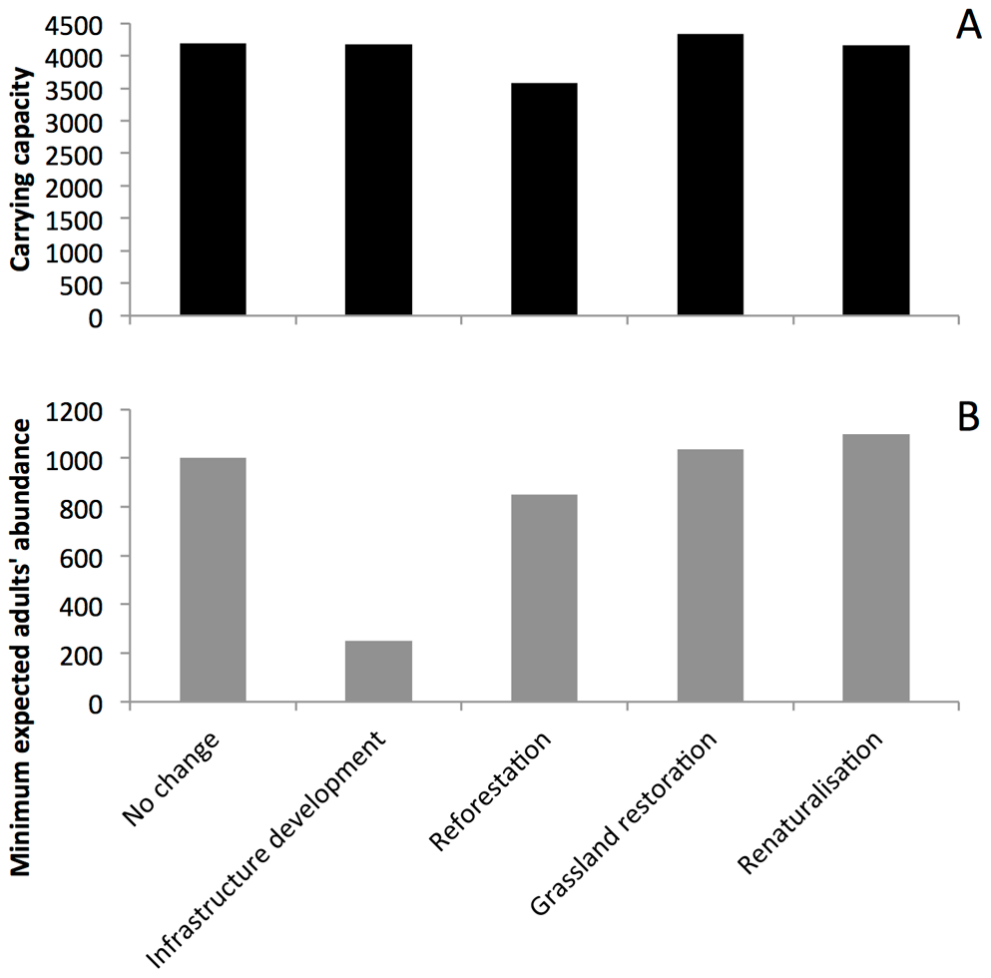
Scenario ranking. (A) Estimated metapopulation carrying capacities of adult individuals for all management scenarios. Estimations were based on habitat suitability and population density. (B) Metapopulation dynamics-based estimations of minimum expected adults’ abundances for all scenarios.

Natterjack toad habitats are distributed throughout the study area, with the biggest patches located in the area of the national park (Figure 1a and b). The habitat network is similar for all management scenarios, comprising mainly small habitat patches (Table 2). In the “no change” scenario the carrying capacity cumulated over all patches is estimated at 4 198 adults. In most other scenarios this value is very similar (Figure 2a). Only in the reforestation scenario carrying capacity decreased by about 15% compared to the “no change” scenario (Figure 2a).

**Table 2:** Number of small (<125), medium (≥125 and <375), and large (≥375 individuals) patches in each scenario.

	Scenario				
Population size	No change	Infrastructure development	Reforestation	Grassland restoration	Renaturalisation
small	18	18	26	18	19
medium	4	4	2	2	4
large	1	1	2	2	1
all	23	23	30	22	24

### Metapopulation Dynamics

Simulations of metapopulation dynamics revealed pronounced differences in minimum expected adults’ abundance (i.e., the average over all simulation runs of the smallest number of adults occurring within the simulated time period; [47]; Figure 2b) and final adults’ abundance (including indicator of variability, Figure S1d) among the alternative scenarios. The “no change” scenario predicted a minimum expected abundance of 1002 adult individuals similar to results from the grassland restoration scenario. The reforestation scenario showed noticeably lower values of minimum expected abundance. The renaturalisation scenario resulted in the highest natterjack toad abundance whereas the infrastructure development scenario resulted in an extremely low abundance (Figure 2b).

### Sensitivity Analysis

Sensitivity analysis of scenario ranking based on total carrying capacity (Figure 2a, Table 3) showed that changing the neighbourhood distance by +/−20% resulted in strong differences in metapopulation structure, especially in the number of small populations (Table S2). Changes (+/−20%) in the density affected both the metapopulation structure and the carrying capacity values (Tables S2 and S3). Sums of preference values for each pair of scenarios summed in rows (Sum+) indicated that the grassland restoration scenario was higher ranked in all 12 cases (Table 3). The reforestation scenario was ranked lowest. Infrastructure development was the second best scenario. The summed column values (Sum−) showed that other scenarios were always better than reforestation.

**Table 3:** Total preference matrix summarising results of carrying capacity sensitivity analysis.

Scenarios	Infrastructure development	Reforestation	Grassland restoration	Renaturalisation	Sum+	Rank
Infrastructure development	0	4	0	4	**8**	**2**
Reforestation	0	0	0	0	**0**	**4**
Grassland restoration	4	4	0	4	**12**	**1**
Renaturalisation	0	4	0	0	**4**	**3**
**Sum**−	**4**	**12**	**0**	**8**		
**Rank**	**2**	**4**	**1**	**3**		

Sensitivity analysis of scenario ranking based on metapopulation dynamics confirmed the robustness of the obtained results (Figure 2b, Table 4). Results from parameter variation provided a preference matrix of metapopulation expected minimum abundance with a column for each scenario and a row for each tested value of the respective parameter (Table S4). The values of the row sums (Sum+) in total the preference matrix indicated that the renaturalisation scenario was preferred 92 out of 93 times (Table 4). The infrastructure development scenario was least preferred. Even though grassland restoration was ranked as the second best scenario, other scenarios were often considered better (1/3 of all cases).

**Table 4:** Total preference matrix summarising results of metapopulation dynamics sensitivity analysis.

Scenarios	Infrastructure development	Reforestation	Grassland restoration	Renaturalisation	Sum +	Rank
Infrastructure development	0	4	0	0	**4**	**4**
Reforestation	27	0	0	0	**27**	**3**
Grassland restoration	31	31	0	1	**63**	**2**
Renaturalisation	31	31	30	0	**92**	**1**
**Sum**−	**89**	**66**	**30**	**1**		
**Rank**	**4**	**3**	**2**	**1**		

Sensitivity analysis of road mortality demonstrated that a 2% change in mortality resulted in the same scenario ranking as the baseline road mortality of 6% (Figure S1).

## Discussion

Our study provides important conclusions regarding the conservation of the natterjack toad and the benefits of scenario ranking using the PVA approach. Taken together our results emphasise the value of species-specific PVA analyses. As illustrated by road mortality in our study, the metric carrying capacity alone is insufficient especially in situations where proposed management actions influence population dynamics. In the common situation where conservationists are confronted with scarce data, parameterisation of the PVA model for natterjack toad is an important result of our work. Following a detailed protocol [48], Tables S1a and S1b comprise a compilation of parameter values that can be useful for other scientists as well as for managers. These tables provide the data necessary to conduct similar analyses in other areas with similar environmental conditions.

Ranking the landscape development scenarios based on the total carrying capacity indicated that most of the proposed management scenarios would have a similar impact on natterjack toad as the “no change” scenario. Only the reforestration scenario showed a difference, specifically this scenario had the lowest estimated carrying capacity. Our results support an earlier analysis of the “no change” scenario [13]. Current habitat conditions, mainly the presence of the national park, allow the preservation of the natterjack toad metapopulation in the study area. However, our study suggests a stronger impact of the infrastructure development scenario on the natterjack toad than former multi-species analyses where more pronounced differences among the alternative management scenarios’ effects were demonstrated for aquatic and forest species [14]. However, habitat requirements of the natterjack toad are complex and coupling breeding habitat with wintering habitat resulted in reduced quantitative differences in carrying capacity. Instead of using existing pond locations as breeding habitat we used habitat in which pond presence is most likely. As mapping of breeding ponds would be possible only for present conditions, this approach allowed us to obtain metapopulation structure for all scenarios.

Ranking of the development scenarios based on metapopulation dynamics resulted in distinct differences among the scenarios. The infrastructure development scenario was the worst option for the conservation of the natterjack toad, and the best option was the renaturalisation scenario (Figure 2). The strong impact of roads on local toad population dynamics most likely explains why the infrastructure development scenario obtained the lowest score. The renaturalisation scenario was the best management option for natterjack toad conservation as eliminating this negative effect of roads (e.g. by providing animal passages) resulted in a high increase in the minimum expected abundance (Table S4). The similarity of the metapopulation dynamics between the grassland restoration and “no change” scenario was most likely caused by the highest resemblance of the management practices in those two scenarios.

Road infrastructure seen as a linear barrier would result in more fragmented metapopulation structure but would not lead to pronounced decrease in habitat availability per se. For amphibians, the most direct influence of roads in the landscape is the increase in mortality [49]. Given their specific breeding behaviour, all pond-breeding amphibians, including natterjack toads, have a high mortality risk due to road crossing [10], [40]. In both of these road-kill studies, natterjack toads were the most common species found among all amphibian and vertebrate species collected. The road-kill hotspots are usually located in suitable habitat patches on local roads with high traffic intensity [10], [40] and many parts of our study area match such a description. The traffic intensity in the Vistula valley exceeds those of the studies mentioned above, reaching to 8000 vehicles per day on minor roads while the major roads reached 32000 vehicles per day in 2004. For several roads in the area the amount of vehicles per day was projected to double by 2010 [50] and in years 2005–2010 increased on average by 22%. Therefore we might expect the amphibian mortalities to be even higher than the values assumed in this study. The analysis of uncertainty in road mortality showed that the scenario ranking did not change as long as there was at least a 2% change in mortality in the infrastructure development and renaturalisation scenarios (Figure S1).

Due to the uncertainty in the estimated road mortality, we do not expect that the PVA carried out in this study will enable accurate predictions of population viability. However, considering the dynamic effects of road mortality on population survival enables us to draw broader conclusions than simply that road mortality has a negative effect on population viability. Despite sparse local data, quantification of these effects and subsequent ranking of landscape development scenarios with different proportions of roadkill mortality is possible.

Considering changes in carrying capacities seems to be a straightforward way to assess changes in available habitat after landscape modifications. However, conservation decisions based upon habitat availability alone can be misleading because they do not include the consequences of demographic and stochastic processes such as dispersal, extinction of small populations, or strategies of habitat use. In particular for amphibians, estimates of carrying capacities provide only poor rankings of management scenarios because of the associated inability to include road mortality in a way that reflects specific habitat use.

Based on our findings we encourage conservation biologists and managers to: combine PVA and habitat modelling; choose appropriate tools that reflect the impact of planned landscape changes; rank and compare the impact of alternative management scenarios produced by PVA; and use real landscape data especially when the species is vulnerable to extinction due to habitat modifications.

## Supporting Information

Figure S1 **Results from sensitivity analysis of road mortality.** Final adults’ abundances and standard deviations are shown for road mortality. Values increased in the infrastructure development scenario and decreased in the renaturalisation scenario by (A) 1%, (B) 2%, (C) 3%, (D) 6% - base level in this study and (E) 9%.
(TIFF)

Table S1 (A) Parameterisation summary based on the ODD protocol and new protocol for PVA. (B) Parameters of the metapopulation model.
(DOC)

Table S2 **Results from sensitivity analysis of predicted metapopulation structure.**
(DOC)

Table S3 **Results from sensitivity analysis of predicted carrying capacity.**
(DOC)

Table S4 **Results from sensitivity analysis of expected minimum abundance.**
(DOC)
