# Supplementary figures and images for: Ranking Landscape Development Scenarios Affecting Natterjack Toad (Bufo calamita) Population Dynamics in Central Poland
Source: PLoS One. 2013 May 29;8(5):e64852. doi: 10.1371/journal.pone.0064852 (PMC3667123; doi:10.1371/journal.pone.0064852)

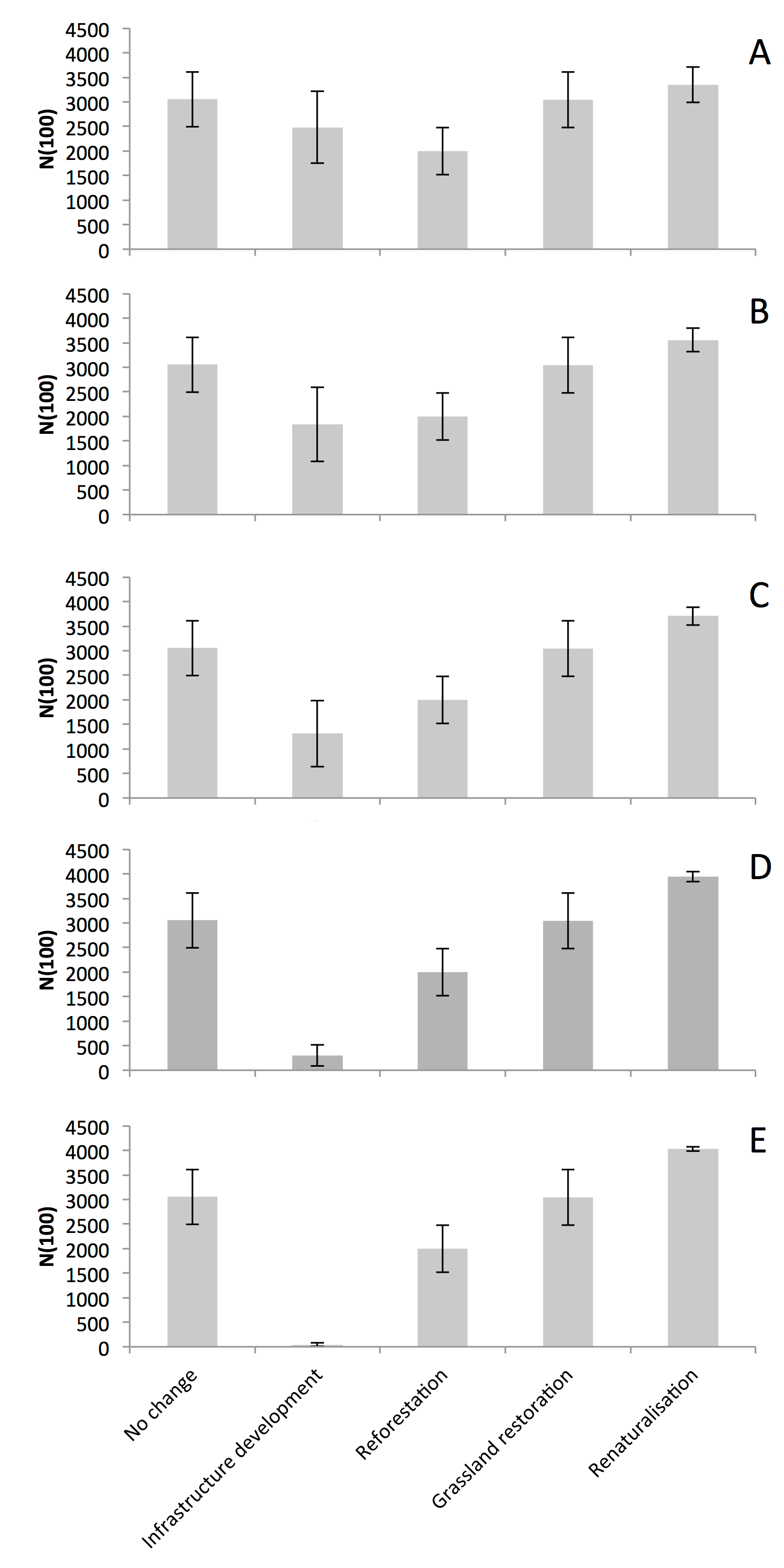

Supplement: Figure S1 — Results from sensitivity analysis of road mortality. Final adults’ abundances and standard deviations are shown for road mortality. Values increased in the infrastructure development scenario and decreased in the renaturalisation scenario by (A) 1%, (B) 2%, (C) 3%, (D) 6% - base level in this study and (E) 9%. (TIFF) [file pone.0064852.s001.tiff]
