# Supplementary material for: Ranking Landscape Development Scenarios Affecting Natterjack Toad (Bufo calamita) Population Dynamics in Central Poland
Source: PLoS One. 2013 May 29;8(5):e64852. doi: 10.1371/journal.pone.0064852 (PMC3667123; doi:10.1371/journal.pone.0064852)
Supplement: Table S1 — (A) Parameterisation summary based on the ODD protocol and new protocol for PVA. (B) Parameters of the metapopulation model. (DOC) [file pone.0064852.s002.doc]

| **Table S1a. Parameterisation summary based on the ODD protocol ^1^ and new protocol for PVA ^2^.** | | | | |
| --- | --- | --- | --- | --- |
| **ODD** | | **DAC-PVA** | | **RAMAS** |
| External to ODD | | Habitat requirements | | Wintering habitat (500m buffer):  - Indefinite pine-birch forest  - Pine thicket  - Juvenile indefinite pine forest  Breeding habitat:  - Wet marsh marigold meadows - 1  - Rich pastures with Cynosurus -1  - Complexes of segetal communities – 0.5  - Oat-grass meadows – 0.5 |
| Purpose | | | | Asses viability of natterjack toad in 5 landscape management scenarios |
| State variables & scales | Entities |  | | Individuals  Patches  Networks of patches |
|  | Spatial units | Spatial entities and units | | Distances [km]  Patch coordinates  Density 50 ind./km^2^ |
|  | Environment | Landscape (spatial collective): | | Landscape extent 135x50km |
|  | Scales: time & space | Temporal units | Time step | year |
|  |  |  | Duration | 100 |
|  |  |  | Repetition | 1000 |
| Processes overview & Scheduling | Processes | Processes I: Population dynamics | Matrix | (see Appendix 1b) |
|  |  |  | Density dependence | Ceiling |
|  |  |  | Group affected | all |
|  |  | Processes III: Dispersal | Dispersal | Implicit |
|  |  |  | D mean | 2km |
|  |  |  | D max | 10km |
|  |  |  | Dispersing group | all |

| Processes overview & Scheduling | Processes | Processes III: Dispersal | Amount of dispersers | Max 20% per connection between two patches |
| --- | --- | --- | --- | --- |
|  |  |  | Mortality | no |
|  |  |  | Formula | Mij = a exp(-Dij^c/b),  a=0.2; b=2; c=1 |
|  | Scheduling | | | Annual sequence:  Population growth  Dispersal |
| Design concept | Emergence | Link between ecological levels | | Patch dynamics emerge from population dynamics and dispersal  Metapopulation dynamics emerge from patch dynamics and dispersal |
|  | Stochasticity | Environmental stochasticity | | Lognormal distribution |
|  |  | Demographic stochasticity | Sex ratio | Binomial distribution |
|  |  |  | Survival | Binomial distribution |
|  |  |  | No. of offspring | Poisson distribution |
|  | Observation | Outputs | | Growth rate |
|  |  |  |  | Final abundance |
|  |  |  |  | Expected minimum abundance |
| Initialization | | | Initial pop size | Same as carrying capacity |
|  |  |  | Carrying capacity | (p1) 11; (p2) 2; (p3) 109; (p4) 67; (p5) 217; (p6) 72; (p7) 252; (p8) 33; (p9) 19; (p10) 16; (p11) 3; (p12) 94; (p13) 208; (p14) 2; (p15) 19; (p16) 2; (p17) 2888; (p18) 20; (p19) 2; (p20) 3; (p21) 5; (p22) 6; (p23) 148 |
|  |  |  | Patch occupancy | All occupied |
|  |  |  | Sex ratio | (See Appendix 1b) |
| Inputs | | | | GIS maps |
| Submodels | | | | landscape |
| External to ODD | Scenario testing / sensitivity / elasticity | | | (See Appendix 1b) |
|  | Outputs | | | (See results) |
|  | Management Implications | Habitat | | Scenario ranking |
|  |  | (Meta)population dynamics | | Scenario ranking |

^1^ Grimm, V., Berger, U., Bastiansen, F., Eliassen, S., Ginot, V., Giske, J., Goss-Custard, J., et al**.,** (2006). A standard protocol for describing individual-based and agent-based models. Ecol. Model. 198(1-2), 115-126.

Grimm, V., Berger, U., DeAngelis, D. L., Polhill, J. G., Giske, J., & Railsback, S. F**.** (2010). The ODD protocol: A review and first update. Ecol. Model. 221(23), 2760-2768.

^2^ Pe’er, G., Matsinos, Y., Johst, K., Franz, K., Turlure, C., Radchuk, V., et al., (2013) . A Protocol for Better Design, Application, and Communication of Population Viability Analyses. Conservation Biology (accepted).

**Table S1b.** **Parameters of the metapopulation model.** Note the three different parameterisations for adult survival rates.

| **Parameter** | | **Value** |
| --- | --- | --- |
| First age of reproduction (F and M) | | 3^rd^ year |
| Maximum breeding age (F and M) | | 7 |
| Sex ratio (F: M) | | 1:1.3 |
| Mating | | polygynous (max. 3 F/M) |
| % adult F breeding | | 90 |
| Mean no. of progeny per breeding F per year | | 5.3 |
| survival of F/M between ages 0 and 1 | | 0.4 / 0.4 |
| survival of F/M between ages 1 and 2 | | 0.45 / 0.45 |
| survival of F/M between ages 2 and 3 | No change, Grassland restoration and Reforestation | 0.6 / 0.55 |
|  | River regulation | 0.54 / 0.49 |
|  | Renaturalisation | 0.66 / 0.61 |
| survival of F/M above age 3 | No change, Grassland restoration and Reforestation | 0.85 / 0.8 |
|  | River regulation | 0.79 / 0.74 |
|  | Renaturalisation | 0.91 / 0.86 |
| Coefficient of variation for fecundities and for survivals | | 0.1 |
