# Supplementary material for: Ranking Landscape Development Scenarios Affecting Natterjack Toad (Bufo calamita) Population Dynamics in Central Poland
Source: PLoS One. 2013 May 29;8(5):e64852. doi: 10.1371/journal.pone.0064852 (PMC3667123; doi:10.1371/journal.pone.0064852)
Supplement: Table S2 — Results from sensitivity analysis of predicted metapopulation structure. (DOC) [file pone.0064852.s003.doc]

**Table S2.** **Results from sensitivity analysis of predicted metapopulation structure.** Results include number of small, medium and large patches in each scenario.

|  |  | **Scenario** | | | |
| --- | --- | --- | --- | --- | --- |
| **Parameter** | **Patch size** | Infrastructure development | Reforestation | Grassland restoration | Renaturalisation |
| distance +20% | small | 10 | 19 | 9 | 11 |
|  | medium | 2 | 2 | 2 | 2 |
|  | large | 2 | 2 | 2 | 2 |
|  | all | 14 | 23 | 13 | 15 |
| distance -20% | small | 40 | 46 | 39 | 39 |
|  | medium | 2 | 2 | 2 | 2 |
|  | large | 1 | 3 | 1 | 1 |
|  | all | 43 | 51 | 42 | 42 |
| density +20% | small | 17 | 26 | 16 | 18 |
|  | medium | 5 | 2 | 4 | 5 |
|  | large | 1 | 2 | 2 | 1 |
|  | all | 23 | 30 | 22 | 24 |
| density -20% | small | 19 | 26 | 19 | 20 |
|  | medium | 3 | 2 | 1 | 3 |
|  | large | 1 | 2 | 2 | 1 |
|  | all | 23 | 30 | 22 | 24 |
