# Supplementary material for: Ranking Landscape Development Scenarios Affecting Natterjack Toad (Bufo calamita) Population Dynamics in Central Poland
Source: PLoS One. 2013 May 29;8(5):e64852. doi: 10.1371/journal.pone.0064852 (PMC3667123; doi:10.1371/journal.pone.0064852)
Supplement: Table S3 — Results from sensitivity analysis of predicted carrying capacity. (DOC) [file pone.0064852.s004.doc]

**Table S3.** **Results from sensitivity analysis of predicted carrying capacity.**

|  | **Scenarios** | | | |
| --- | --- | --- | --- | --- |
| **Parameter** | Infrastructure development | Reforestation | Grassland restoration | Renaturalisation |
| dispersal distance +20% | 4182 | 3580 | 4338 | 4161 |
| dispersal distance -20% | 4185 | 3580 | 4342 | 4163 |
| density +20% | 5018 | 4298 | 5208 | 4998 |
| density -20% | 3346 | 2862 | 3471 | 3331 |
