# Supplementary material for: Ranking Landscape Development Scenarios Affecting Natterjack Toad (Bufo calamita) Population Dynamics in Central Poland
Source: PLoS One. 2013 May 29;8(5):e64852. doi: 10.1371/journal.pone.0064852 (PMC3667123; doi:10.1371/journal.pone.0064852)
Supplement: Table S4 — Results from sensitivity analysis of expected minimum abundance. (DOC) [file pone.0064852.s005.doc]

**Table S4**. Results from sensitivity analysis of expected minimum abundance.

|  | **Scenarios** | | | |
| --- | --- | --- | --- | --- |
| **Parameter** | Infrastructure development | Reforestation | Grassland restoration | Renaturalisation |
| initial abundance +20% | 770 | 1968 | 2614 | 3245 |
| initial abundance -20% | 684 | 1921 | 2573 | 3212 |
| all dispersal rates +20% | 747 | 1894 | 2575 | 3232 |
| all dispersal rates - 20% | 768 | 2039 | 2660 | 3267 |
| breeding success + 20%^1^ | 2197 | 2564 | 3227 | 3487 |
| breeding success -20% | 57 | 563 | 934 | 2680 |
| offspring number +20% | 2788 | 2762 | 3472 | 3600 |
| offspring number -20% | 3 | 54 | 113 | 1335 |
| dispersal rate +20% | 762 | 1882 | 2557 | 3231 |
| dispersal rate -20% | 774 | 2025 | 2636 | 3267 |
| mean disp. distance +20% | 734 | 1665 | 2493 | 3167 |
| mean disp. distance -20% | 797 | 2145 | 2713 | 3306 |
| max disp. distance +20% | 776 | 1900 | 2618 | 3249 |
| max disp. distance -20% | 773 | 1991 | 2604 | 3256 |
| 1 stage survival rate + 20%^2^ | 2877 | 2824 | 3549 | 3662 |
| 1 stage survival rate -20% | 3 | 49 | 109 | 1320 |
| 2 stage survival rate +20% | 2903 | 2843 | 3557 | 3663 |
| 2 stage survival rate -20% | 3 | 52 | 108 | 1298 |
| 3 stage survival rate +20% | 2409 | 2688 | 3377 | 3584 |
| 3 stage survival rate -20% | 37 | 333 | 594 | 2242 |
| 4 stage survival rate +20% | 1950 | 2501 | 3158 | 3414 |
| 4 stage survival rate -20% | 121 | 772 | 1264 | 2664 |
| 5 stage survival rate +20% | 1471 | 2352 | 2985 | 3357 |
| 5 stage survival rate -20% | 279 | 1265 | 1905 | 2947 |
| 6 stage survival rate +20% | 1076 | 2167 | 2796 | 3300 |
| 6 stage survival rate -20% | 523 | 1693 | 2346 | 3126 |
| adult stages surv. rate +20% | 3358 | 3045 | 3792 | 3731 |
| adult stages surv. rate -20% | 0.4 | 3 | 12 | 170 |
| K +20% | 870 | 2379 | 3178 | 3939 |
| K -20% | 590 | 1517 | 2019 | 2538 |
| without national park^3^ | 55 | 156 | 455 | 714 |

Breeding success equals 100% (initial value was 90%).

^2^ For each stage both male and female survival rates were changed.

^3^ Big patches from the national park area were excluded from the simulation.
